# A polar nature of benzoic acids extrusion from nitroalkyl benzoates: DFT mechanistic study

**DOI:** 10.1007/s00894-015-2592-6

**Published:** 2015-02-25

**Authors:** Radomir Jasiński, Agnieszka Kącka

**Affiliations:** Institute of Organic Chemistry and Technology, Cracow University of Technology,, Warszawska 24, 31-155 Cracow, Poland

**Keywords:** DFT study, Mechanism, Nitrocompounds, Thermal elimination

## Abstract

Using DFT calculations at various theory levels, quantum-chemical simulations of decomposition paths were performed for a series of nitroalkyl benzoates. It was discovered, that these reactions proceed via polar, but one-step mechanism. It turned out that depending on the nature of the substituent in the ester molecule and on medium polarity, the studied reactions may take place via transition states with varied synchronicity — from E1-like structures, to E1cb-like structures. A purely ionic, two-stage mechanism was not identified in any of the cases.

**Graphical Abstract:**
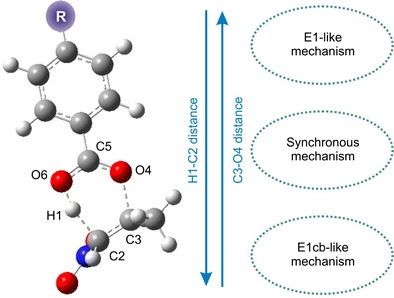
Benzoic acids extrusion from nitroalkyl benzoates

Benzoic acids extrusion from nitroalkyl benzoates

## Introduction

Conjugated nitroalkenes are very valuable precursors in organic synthesis. They are used, e.g., in syntheses of many four-, five-, and six-membered carbo- and heterocycles in cycloaddition reactions [1–4]. The presence of a nitro group adjacent to the vinyl moiety activates it strongly in reactions with nucleophilic reagents on one side, and on the other, enables introduction of a nitro group to the final products, characterized by an exceptionally wide spectrum of potential transformation directions [4–8]. In practice, it results in the possibility of further functionalization.

**Scheme 1:**
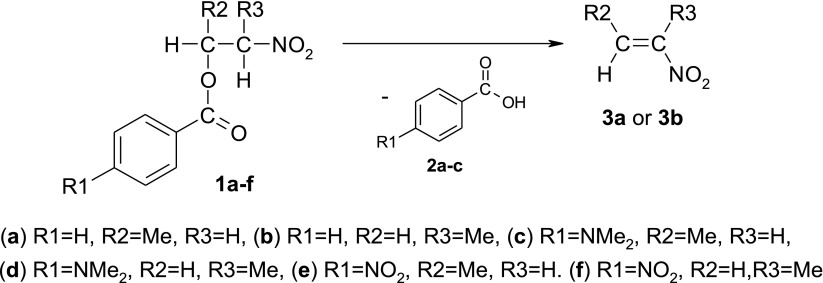
Benzoic acids extrusion from nitroalkyl benzoates

The most universal strategies for their synthesis are based on the decomposition of esters of appropriate β-nitroalcohols [4]. These may be, e.g., benzoic acid esters [9], which are easy to synthesize and isolate (e.g., Scheme 1).

**Scheme 2:**
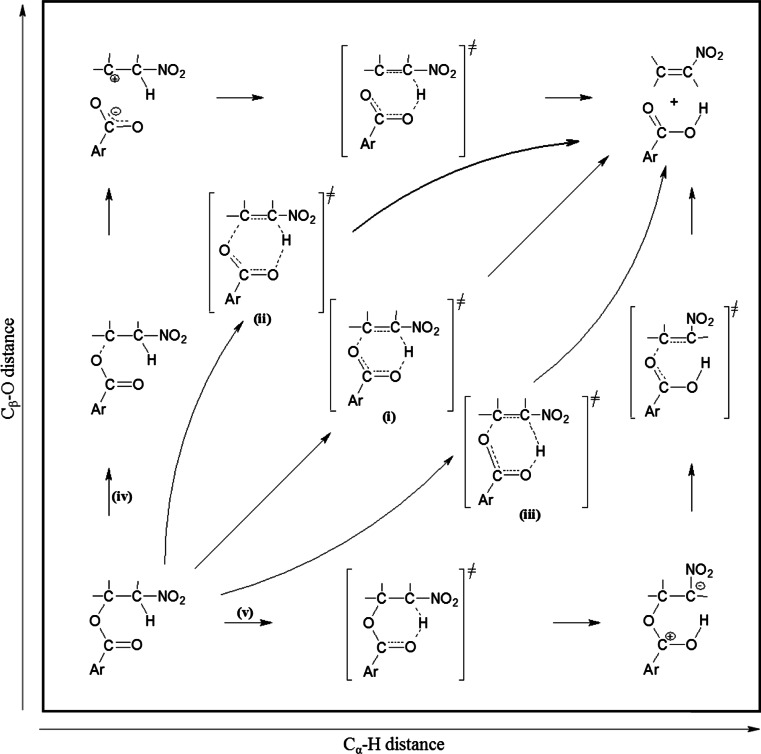
Five theoretically possible reactions mechanisms for decomposition of nitroalkyl benzoates

It is accepted, in general, that esters thermolysis takes place according to a synchronous, “pericyclic” mechanism, via a six-membered transition complex. It should be underlined however, that several atypical mechanisms have been discovered recently about reactions, which earlier without any doubt were considered as synchronous and “pericyclic”: zwitterionic, stepwise [2 + 3] cycloadditions [10–14], extremely asynchronous nitrous acid extrusion [15, 16], thermal decomposition of fluoronitroazoxy compounds [17], as well as multi-step reactions between dienes and ethylenic dienophiles which carry out via [3.3]-sigmatropic shift stage instead of according to typical Diels-Alder mechanism [18–21]. Next, *Domingo* [22] generally undermines “pericyclic” notion for several organic reactions. It is significant that many of these anomalous mechanisms have been implemented in relation to nitrocompounds. In consequence, general examination of mechanistic aspects of nitroalkyl carboxylates decompositions process is necessary. Disturbances in the electron-density redistribution within transition states of benzoic acid extrusion reactions will be certainly stimulated by the electron-withdrawing character of nitrogroup. Therefore, in the case of nitroalkyl benzoates, not one, but five theoretically possible reactions mechanisms should be considered (Scheme 2): (i) ideal “pericyclic” mechanism, (ii) asynchronous E1-like mechanism, (iii) asynchronous E1cb-like mechanism as well as (iv,v) purely ionic mechanisms.

With these concerns in mind, in this work, we have initiated DFT mechanistic studies of model decomposition reactions of selected nitroalkyl benzoates (Scheme 2). In particular, we have performed simulations of reaction paths for processes involving benzoates that contain substituents with various donor-acceptor power in the phenyl ring. We analyzed these reactions both in gaseous phase and in the simulated presence of dielectric media.

## Computational methods

All calculations reported in this thesis were performed on an SGI-Altix 3700 computer in the CYFRONET regional computational center in Cracow. Hybrid functional B3LYP with the 6-31G(d) basis set included in the GAUSSIAN 09 package [23] was used. Recently published reports show that the same theoretical level was used, e.g., for the analysis of chemical properties of nitro-functionalized compounds [12, 15, 17, 24, 25] including thermal decomposition process [15, 17]. In addition, similar simulations using more advanced B3LYP/6-31G(d,p), B3LYP/6-31 + G(d) as well as B3LYP/6-311G(d) theoretical levels were performed. Optimizations of the stable structures were performed with the Berny algorithm, whereas the transition states were calculated using the QST2 procedure followed by the TS method. Stationary points were characterized by frequency calculations. All reactants, and products had positive Hessian matrices. All transition states showed only one negative eigenvalue in their diagonalized Hessian matrices, and their associated eigenvectors were confirmed to correspond to the motion along the reaction coordinate under consideration. For all reactions, intrinsic reaction coordinate (IRC) calculations were performed to connect previously computed transition structures (TS) with suitable minima. For the calculations of solvent effect on the reaction paths the polarizable continuum model (PCM) [26] in which the cavity is created via a series of overlapping spheres was used. Charge global electron density transfer (GEDT) [22] was calculated according to the formula:

$$ \mathrm{GEDT}=-\varSigma {\mathrm{q}}_{\mathrm{A}} $$

where q_A_ is the net charge and the sum is taken over all the atoms of substructure. The same calculation methodology was applied to solutions as to the gas phase. Results are collected in Tables 1, 2, and 3.

**Table 1:** Kinetic and thermodynamic parameters for thermal decomposition of nitroalkyl benzoates 1a–f according to B3LYP/6-31G(d) calculations (T = 298 K; ΔH, ΔG in kcal mol^−1^; ΔS in cal mol^−1^ K^−1^)

Ester	Solvent (ε)	Transition	ΔH	ΔG	ΔS
1a	Gas phase	1a → TS	34.2	35.0	−2.7
	(1.0000)	1a → 2a + 3a	12.4	0.2	40.9
	Toluene	1a → TS	33.3	34.4	−3.6
	(2.3741)	1a → 2a + 3a	10.9	−1.0	40.1
	Water	1a → TS	32.3	33.5	−4.2
	(78.3553)	1a → 2a + 3a	9.5	−2.3	39.6
1b	Gas phase	1b → TS	40.6	41.5	−3.1
	(1.0000)	1b → 2a + 3b	14.6	2.7	39.9
	Toluene	1b → TS	39.8	40.7	−3.1
	(2.3741)	1b → 2a + 3b	13.5	1.6	40.0
	Water	1b → TS	38.3	39.0	−2.3
	(78.3553)	1b → 2a + 3b	12.3	0.4	39.8
1c	Gas phase	1c → TS	33.3	34.5	−4.0
	(1.0000)	1c → 2c + 3a	12.2	1.6	35.5
1d	Gas phase	1d → TS	39.5	40.9	−4.7
	(1.0000)	1d → 2c + 3b	14.4	4.1	34.3
1e	Gas phase	1e → TS	34.9	35.4	−1.7
	(1.0000)	1e → 2e + 3a	12.3	0.0	41.4
1f	Gas phase	1f → TS	41.5	42.5	−3.6
	(1.0000)	1f → 2e + 3b	14.3	2.8	38.7

**Table 2:** Kinetic and thermodynamic parameters for thermal decomposition of nitroalkyl benzoates 1a, b according to B3LYP/6-31G(d,p), B3LYP/6-31+G(d) and B3LYP/6-311G(d) calculations (T = 298 K; ΔH, ΔG in kcal mol^−1^; ΔS in cal mol^−1^ K^−1^)

Ester	Theory level	Transition	ΔH	ΔG	ΔS
1a	B3LYP/	1a → TS	31.8	32.5	−2.6
	6-31G(d,p)	1a → 2a + 3a	9.8	−2.5	41.3
	B3LYP/	1a → TS	33.2	34.1	−3.1
	6-31+G(d)	1a → 2a + 3a	8.6	−3.5	40.7
	B3LYP/	1a → TS	34.6	35.3	−2.2
	6-311G(d)	1a → 2a + 3a	10.1	−2.2	41.4
1b	B3LYP/	1b → TS	38.1	39.1	−3.2
	6-31G(d,p)	1b → 2a + 3b	11.8	0.0	39.8
	B3LYP/	1b → TS	40.3	41.1	−2.7
	6-31+G(d)	1b → 2a + 3b	11.8	−0.1	40.0
	B3LYP/	1b → TS	41.2	42.0	−2.6
	6-311G(d)	1b → 2a + 3b	12.7	0.6	40.3

**Table 3:** Electronic and geometrical characteristics of key structures of thermal decomposition of nitroalkyl benzoates 1a–f according to B3LYP/6-31G(d) calculations

Dielectric constants of reaction environment ε	Reaction	Structure	Interatomic distances [Ǻ]						GEDT [e]	Dipole moment μ [D]
			H1-C2	C2-C3	C3-O4	O4-C5	C5-O6	O6-H1		
1.0000	1a → 2a + 3a	1a	1.091	1.528	1.450	1.359	1.217	2.398	0.14	3.18
		TS	1.503	1.430	1.810	1.272	1.290	1.128		6.72
		2a + 3a		1.333		1.215	1.359	0.975		
1.0000	1b → 2a + 3b	1b	1.091	1.525	1.434	1.363	1.216	2.547	0.18	2.39
		TS	1.543	1.431	1.750	1.273	1.291	1.107		6.92
		2a + 3b		1.332		1.215	1.359	0.975		
1.0000	1c → 2c + 3a	1c	1.086	1.529	1.445	1.366	1.220	2.381	0.19	4.85
		TS	1.561	1.434	1.764	1.278	1.297	1.094		10.38
		2c + 3a		1.333		1.218	1.364	0.975		
1.0000	1d → 2c + 3b	1d	1.089	1.525	1.430	1.371	1.219	2.690	0.22	4.37
		TS	1.601	1.435	1.714	1.279	1.298	1.076		10.04
		2c + 3b		1.332		1.218	1.364	0.975		
1.0000	1e → 2e + 3a	1e	1.091	1.527	1.454	1.353	1.215	2.493	0.08	5.76
		TS	1.452	1.426	1.865	1.269	1.286	1.164		5.57
		2e + 3a		1.333		1.213	1.355	0.975		
1.0000	1f → 2e + 3b	1f	1.090	1.524	1.438	1.357	1.214	2.730	0.13	5.36
		TS	1.494	1.426	1.793	1.270	1.286	1.137		4.93
		2e + 3b		1.332		1.213	1.355	0.975		
2.3741	1a → 2a + 3a	1a	1.086	1.528	1.451	1.357	1.218	2.409	0.18	3.52
		TS	1.551	1.436	1.771	1.273	1.293	1.100		8.34
		2a + 3a		1.334		1.217	1.356	0.975		
2.3741	1b → 2a + 3b	1b	1.089	1.525	1.436	1.360	1.217	2.729	0.22	2.56
		TS	1.602	1.437	1.714	1.273	1.294	1.077		8.67
		2a + 3b		1.332		1.217	1.356	0.975		
78.3553	1a → 2a + 3a	1a	1.086	1.529	1.454	1.354	1.220	2.427	0.23	3.93
		TS	1.625	1.443	1.726	1.273	1.297	1.066		10.43
		2a + 3a		1.336		1.219	1.353	0.976		
78.3553	1b → 2a + 3b	1b	1.089	1.525	1.438	1.358	1.219	2.740	0.27	2.81
		TS	1.690	1.443	1.679	1.273	1.299	1.044		11.00
		2a + 3b		1.333		1.219	1.353	0.976		

## Results and discussion

### Energy profiles

**Fig. 1:**
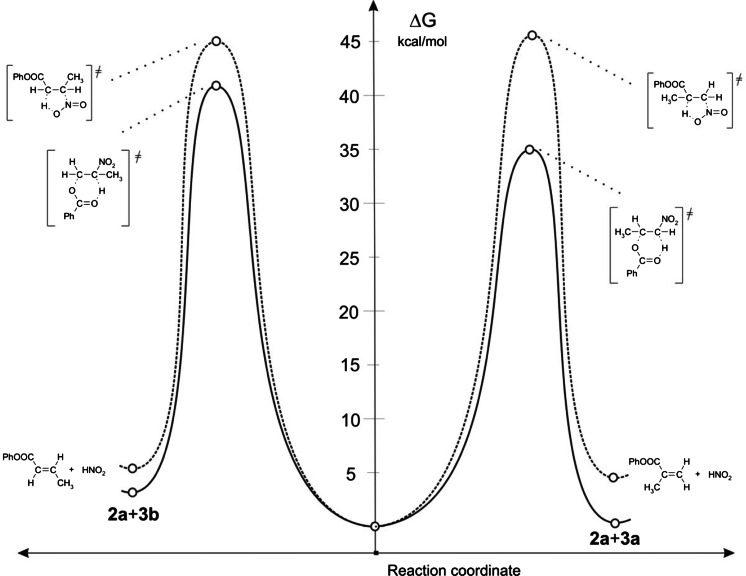
Gibbs free energy profiles for thermal decomposition of esters 1a and b in gas phase according to B3LYP/6-31G(d) calculations (T = 298 K)

B3LYP/6-31G(d) calculations showed that conversion of esters 1a and b into respective nitroalkenes in gaseous phase proceeds according to a one-step mechanism. In both cases, between the minima for substrates and respective products (Fig. 1) exists only one transition state (TS). This is confirmed by IRC calculations. All attempts to find the ionic intermediate on reaction paths were not successful.

However, reaching this critical point by the reacting system may require a different energy requirement to be met (Table 1). In particular, the decomposition process of nitrobenzoate 1a requires crossing the activation barrier of ΔG = 35 kcal mol^−1^. Fundamentally, it is associated with increasing enthalpy of reaction system. On the other hand, entropy of reaction system only slightly changes. This is typical for elimination reactions which lead via high ordered transition states [27]. In case of an analogous reaction involving nitrobenzoate 1b entropy of activation is also low, but the activation barrier (determined by ΔH value) is greater than 41.5 kcal mol^−1^. This means that the presence of a substituent in the vicinal position in relation to the nitro group makes extrusion of a molecule of benzoic acid faster. Presumably, this is a result of higher substituents crowd on β carbon atom of nitroalkyl moiety. In consequence, this accelerates dissociation of -O-C(O)-Ph group.

Subsequently, we have performed simulations of theoretically probable paths of nitrous acid extrusion from 1a and b. It was found, that these reactions proceed via one step, Cope-like mechanism similar to described earlier thermal decomposition of the product derived from 3-nitro-2-(trifluoromethyl)-2H-chromene and 2-(1-phenylpropylidene)malononitrile [15]. It should be noted however, that nitrous acid extrusion process should be considered forbidden from kinetic point of view (activation barriers equal 45.7 and 44.9 kcal mol^−1^ for decomposition of 1a and b respectively).

A similar image of these reactions in gaseous phase is supplied by calculation on higher theory levels (B3LYP/6-31G(d,p), B3LYP/6-31+G(d), B3LYP/6-311G(d)). In particular, all performed simulations clearly indicate a one-step reaction mechanism, and activation barriers along individual paths do not differ significantly from those obtained on the basis of B3LYP/6-31G(d) calculations. In every case, ester decomposition 1a takes place much more easily than that of 1b (Table 2).

B3LYP/6-31G(d) calculations also make it possible to shed some light on the influence of the substituent in the leaving group on the course of reaction. It turned out that regardless of the nature of substituent in the benzene ring, esters of both 1-nitropropane-1-ol and 2-nitropropane-1-ol will undergo decomposition according to a one-step mechanism. It must also be noted that electrodonating groups (e.g., NMe_2_) will lower the activation barrier of the decomposition process, while electroaccepting groups (e.g., NO_2_) will make the process more difficult.

The further course of quantum-chemical studies also included analysis of solvent influence on reaction kinetics. It turned out that a polarity increase of the reaction medium facilitates lowering of the activation barrier. It does not change, however, the mechanism of carboxylic acid cleavage from the parent ester. In all cases (even extremely polar aqueous environment), all attempts to find alternatively, two-step reaction paths were not successful.

### Transition structures

**Fig. 2:**
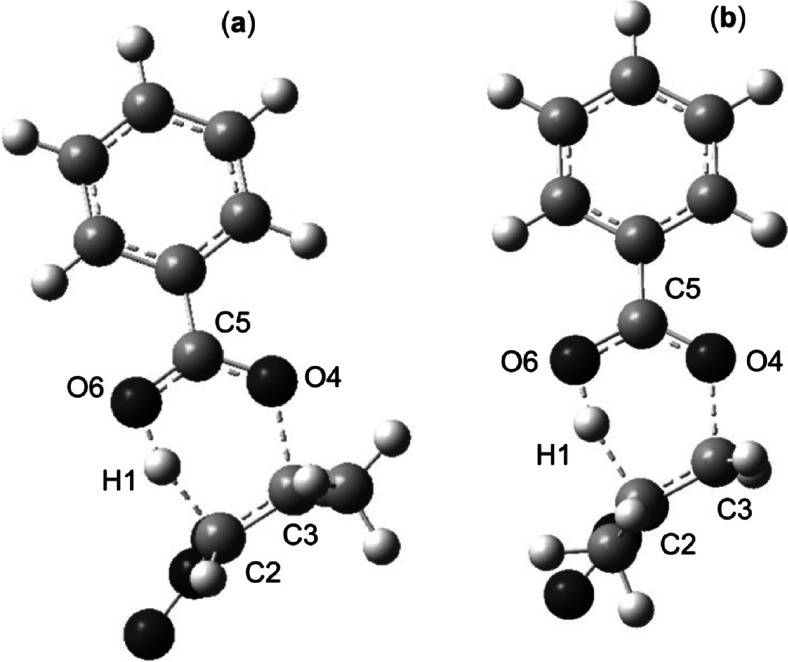
Transition states for thermal decomposition reactions of esters 1a (**a**) and b (**b**) in gas phase according to B3LYP/6-31G(d) calculations (T = 298 K)

Studies on the transition state structure (TS) were started with a reaction involving esters 1a and b. It turned out that both of these TSs have a six-membered structure (Fig. 2). A new bond is formed within both of these structures (H1-O6) and at the same time, the nature of C2-C3 and C5-O4 bonds changes (the bond gains features characteristic for a double bond) and C5-O6 (the bond gains features characteristic for a single bond). Simultaneously, H1-C2 and C3-O4 bonds become broken. Loosening of these bonds is, however, significantly different.

The H1-C2 in the case of reaction of 1a → 2a + 3a is broken more slowly than in the case of reaction 1b → 2a + 3b. In turn, the C3-O4 bond of TS of reaction 1a → 2a + 3a is broken faster than in the case of reaction 1b → 2a + 3b. This means that the energetically relatively more favorable transition state of the 1a → 2a + 3a process has a more profound, asynchronous character.

Subsequently to asynchronicity of bonds loosening, asynchronous redistribution of electron density asynchrony is observed. This is evidenced by GEDT index values (see Table 3). Moreover, GEDT values as well as dipole moments confirmed polar nature of TSs. In consequence — according to *Domingo* terminology [22] — they cannot be considered as “pericyclic”. Next, we have re-optimized transition state structures using UB3LYP/6-31G(d) theory level. It was found, that both TSs have non-biradicaloid character. This is confirmed by < S2 > values which in both cases equal 0.00.

It must be noted that the geometric parameters of the studied TSs obtained using higher theory levels (B3LYP/6-31G(d,p), B3LYP/6-31+G(d), B3LYP/6-311G(d)) are practically identical.

B3LYP/6-31G(d) calculations also provided us with information about the influence of the substituent in the leaving group on the TS structure. It turned out that both in the case of 1-nitropropane-1-ol and 2-nitropropane-1-ol esters, the presence of an electrodonating substituent facilitates an increase in TS synchronicity, whilst the presence of an electroaccepting substituent facilitates an increase in TS asynchronicity. The influence of the nature of the substituent on the symmetry of transition complex is in general decidedly too weak to enforce a change of the reaction mechanism to a two-step one. On the other hand, substituent nature stimulated asynchronicity of electron density redistribution. In particular, TSs of reaction involving dimethylamino-substituted esters are characterized by relatively higher GEDT values, whereas TSs of reaction involving nitro-substituted esters — relatively lower. However, in any case this process is ideal synchronous (GEDT ≠ 0.00e).

Finally, we have also analyzed the influence of the solvent on the structure of TSs. It turned out that more polar medium facilitates a faster loosening of the H1-C2 bond. At the same time, it makes breaking the C3-C4 bond more difficult. Asynchronicity of TSs increases in an extremely polar, aqueous medium. In consequence, TSs for reactions 1a → 2a + 3a and reaction 1b → 2a + 3b under these conditions should be interpreted rather as similar to the E1cb-like type. It should be noted, that increasing of environment polarity stimulated more polar nature of TSs. This is confirmed by GEDT values, which reach even above 0.22e. However, it is not sufficient (even in extremely polar, aqueous solution) to generate evidence of ionic structures.

## Conclusions

DFT calculations — regardless of theory level — indicate a one-step mechanism of thermal decomposition of nitroalkyl benzoates. However, depending on the nature of the substituent in the ester molecule and on medium polarity, the studied reactions may take place via transition state structures with varied synchronicity — from E1-like structures, through a rather synchronous model, to E1cb-like structures. It must be noted that a purely ionic, two-stage mechanism was not identified in any of the cases. Reaction kinetics may influence — albeit to a limited extent — the nature of the substituent and polarity of the reaction medium.
